# Monoconjugation of Human Amylin with Methylpolyethyleneglycol

**DOI:** 10.1371/journal.pone.0138803

**Published:** 2015-10-08

**Authors:** Tháyna Sisnande, Luiz Henrique Guerreiro, Raquel R. Braga, Luana Jotha-Mattos, Luiza C. S. Erthal, Priscilla Tinoco, Bruno M. Ferreira, Luís Maurício T. R. Lima

**Affiliations:** 1 Federal University of Rio de Janeiro–UFRJ, CCS, Bss24, Ilha do Fundão, 21941–590, Rio de Janeiro, RJ, Brazil; 2 Department of Chemistry, Institute of Exact Sciences, Rural Federal University of Rio de Janeiro—Universidade Federal Rural do Rio de Janeiro, Rodovia BR 465, km 7, CEP: 23890–000, Seropédica, RJ, Brazil; 3 Laboratory for Macromolecules (LAMAC-DIMAV), Brazilian National Institute of Metrology, Quality and Technology—INMETRO, Av. N. Sa. das Graças, 50—Xerém, Duque de Caxias-RJ, 25250–020, Rio de Janeiro, Brazil; 4 National Institute of Science and Technology for Structural Biology and Bioimaging (INBEB-INCT), Federal University of Rio de Janeiro, Rio de Janeiro 21941–590, Brazil; University of Lancaster, UNITED KINGDOM

## Abstract

Amylin is a pancreatic hormone cosecreted with insulin that exerts unique roles in metabolism and glucose homeostasis. The therapeutic restoration of postprandial and basal amylin levels is highly desirable in diabetes mellitus. Protein conjugation with the biocompatible polymer polyethylene glycol (PEG) has been shown to extend the biological effects of biopharmaceuticals. We have designed a PEGylated human amylin by using the aminoreactive compound methoxylpolyethylene glycol succinimidyl carbonate (mPEGsc). The synthesis in organic solvent resulted in high yields of monoPEGylated human amylin, which showed large stability against aggregation, an 8 times increase in half-life *in vivo* compared to the non-conjugated amylin, and pharmacological activity as shown by modulation of cAMP production in MCF–7 cell line, decrease in glucagon and modulation of glycemia following subcutaneous administration in mice. Altogether these data reveal the potential use of PEGylated human amylin for the restoration of fasting amylin levels.

## Introduction

Amylin (also known as islet amyloid polypeptide, or IAPP) is a pancreatic peptide cosecreted with insulin from the β-cells in the Langerhans islets [1]. Amylin is an unique hormone exerting a complex network of physiologic functions, such as regulation of β-, α- and δ- cells secretion, inhibiting glucagon, somatostatin and β-adrenoceptor-induced insulin secretion [2–5], gastric emptying [6,7], decrease in glycemia [4,8], increase in lactate production, decrease in glycogen synthesis and glucose uptake in muscle [9,10].

Amylin was discovered from amyloid deposits in pancreas [11,12]. Amyloid deposits in pancreas of diabetic individuals has long been shown since the seminal work of Opie [13], and both toxic oligomers and amyloid fibrillar aggregates occurs in concurrence with loss in beta-cell activity [14–17]. Despite the intensive research in the field, the molecular mechanism for the formation of amyloid deposits in pancreas is not clearly understood. Some factors that might influence the amylin aggregation are modulation of IDE [18,19], interaction with lipid interfaces [20,21], unbalanced interaction with insulin [22,22–25,25,26], A-β [27] or metals [28–31].

The progressive dysfunction of β-cells in T2DM results in the decrease in levels of both insulin and amylin [32], leading to the need for hormone therapy at advanced stages of the disease [33]. Along with insulin, therapeutic replacement of amylin, as originally suggested [34], is nowadays recommended for a more tight control of glycemia in individuals with either type 1 or type 2 diabetes mellitus (T1DM or T2DM, respectively) [10,33,35–41]. The dissimilar levels of expression, metabolism and distribution does not allows the use of a insulin:amylin concentration ratio for clinical purposes [32,42,43], as well as a lack in the correlation between insulin sensitivity and β-cell function [44]. In fact, a correlation between circulating amylin concentration and HOMA might display a more clinical realistic correlation in the diagnostic and monitoring scenario.

The therapeutic use of human amylin has not been possible due to its limited solubility in aqueous milieu [45], which result in amylin oligomer and amyloid fibrils. This feature is not limited human amylin, since despite the higher solubility of proline-rich amylin variants compounds [46], they may also result in amyloid aggregation [47].

The proline effect on the enhancement of amylin stability have inspired the development of a triple-proline variant of the human amylin (Pro^25,28,29^) pramlintide and patent [48,49], named pramlintide, which has been made available in the US since 2005. Pramlintide is used by subcutaneous (s.c.) injection along with insulin by mealtime, mimicking the post-prandial levels of amylin. Thought pramlintide brought remarkable benefits for management of diabetes [50,51], diabetic individuals still face limitations: i) amylin must be injected separately from insulin [52] since its has been observed that amylin interacts with insulin [22,53,54]; ii) restoration of the basal amylin level is not achieved with the products currently available; iii) there is no therapeutics based on homologous human amylin.

**Fig 1:**
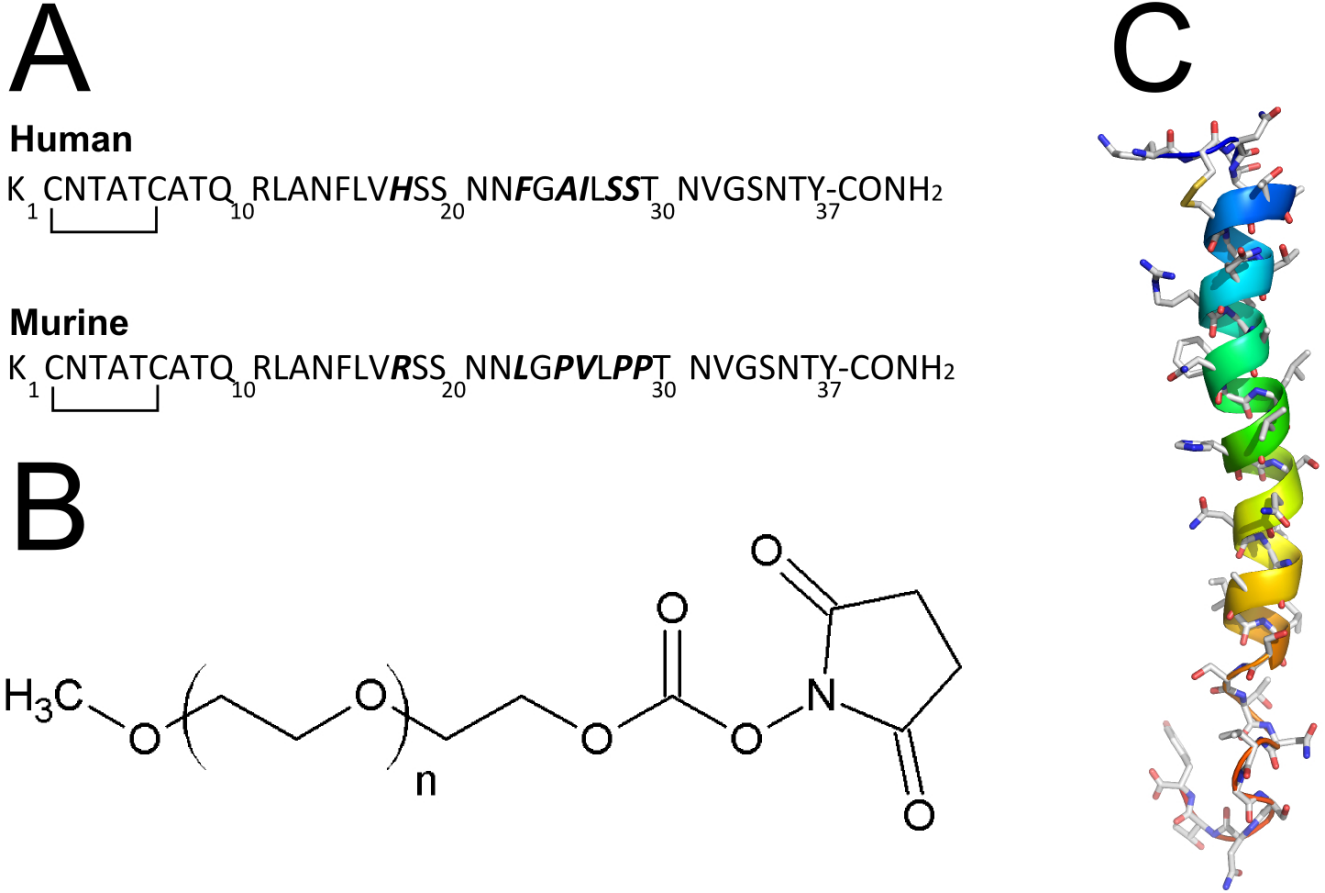
Amylin and PEG structures. **a)** Aminoacid sequence of the human and murine amylin. In bold are the different aminoacid. Notice the similarity in sequence from aminoacid 1 to 17. The N-terminus is the Lys1, which comprises the α- and ε-aminogroup, the two unique primary aminogroups in amylin targeted for PEGylation. **b)** Chemical structure of the methoxyl PEG N-hydroxylsuccinimide (NHS) carbonate. **c)** Structure of the human amylin. Human amylin (from NMR structure, PDB ID 2KB8) is represented in ribbons colored from aminoacid 1 (blue) to 37 (red). Notice the two aminogroups at the top left side of the representation.

In order to address the solubility and agglomerating issues of human amylin, we have designed a strategy based on the conjugation with the highly soluble, biocompatible polymer polyethylene glycol (PEG). The introduction of a PEG moiety to murine amylin resulted in a sustained effect *in vivo* longer than the non-conjugated hormone [55,56]. Human and murine amylin have equivalent sequence from aminoacids 1 to 17 (**Fig 1A**), and they both have only two primary amine in their chain: the α and ε aminogroups of the Lys_1_. We targeted the PEGylation of this aminoacid by using the methoxy polyethylene glycol (mPEG) succinimidyl carbonate (mPEGsc; **Fig 1B**), which display reactivity preferably for the amino group thought not exclusively [55,56]. In this work we present the results of the synthesis, purification, physico-chemical and pharmacological evaluation of the PEGylated human amylin.

## Materials and Methods

### Reagents

Carboxy-amidated amylin, with a disulfide bridge between C2 and C7 was obtained from Genemed Synthesis Inc (Inc (San Antonio, TX)), both murine (CAS 124447-81-0]) and human (CAS 122384-88-7]). Stock human amylin solutions were prepared at 10 mg/mL in DMSO. Methoxyl polyethylene glycol succinimidyl carbonate with average molecular numbers 5 kDa (mPEGsc5k; polydispersity = 1.08, as informed by the manufacturer) was purchased from Nanocs (USA). All other reagents were of analytical grade.

### Amylin PEGylation and purification

Typically the reaction is conducted at 1, 2 or 5 mg/mL human amylin (final concentration), by mixing human amylin (from stock at 10 mg/mL in DMSO) with the required amount of mPEGsc5k (from stock solution in DMSO), phosphate buffered saline (10xPBS stock: 81 mM Na_2_HPO_4_, 18 mM KH_2_PO_4_, 27 mM KCl, 1370 mM NaCl, pH 7.4) to provide a final dilution to 1/100 of the reaction volume, and sufficient amount of DMSO to achieve the desired volume and allowed to react at the indicated temperature. The reaction was quenched by either addition of equal volume of 30% CH_3_CN / 70% water/0.1% TFA followed by immediate C18-RP-HPLC purification or characterization by mass spectrometry, or quenched with the sodium dodecyl-sulfate polyacrylamide gel electrophoresis (SDS-PAGE) loading buffer [57–60].

The reaction was then separated in a Kromasil C18 column (250 x 10 mm, particle size 5 μm) with a flow rate of 4 mL/min, in a Jasco LC–2000 HPLC (Jasco Inc, USA) The gradient was as follows: 15 min linear gradient of CH_3_CN in water containing 0.1% (v/v) TFA, progressing from 30% (after 5 min at 30% CH_3_CN) to 70%. The purification was monitored by following the absorbance at 220 nm. The samples, pooled or separated, were lyophilized, and dissolved with water for immediate use as indicated elsewhere. The amylin concentration was accessed by absorbance at 280 nm by using 1,615 M^-1^cm^−1^ as extinction coefficient. Attempts to use colorimetric assays for the quantification of amylin, such as Bradford, Lowry, BCA or Qubit, did not provide consistent results.

### Electrophoretic analysis of the PEGylation products

Both the PEGylation reaction and products were analyzed by a 18% SDS-PAGE [57–60]. Typically an aliquot corresponding to no less than 50 μg peptide was used per lane. The PAGE were stained with Coomassie brilliant blue G–250 for detection of the peptide moiety, followed by staining with barium-iodine protocol for detection of the PEG moiety [57]. Analysis of the digitized PAGE was performed with ImageJ [61].

### Kinetic analysis of the amylin PEGylation reaction

The PEGylation reaction was followed by a derivatization of the remaining primary amines in amylin with fluorescamine, as described in detail elsewhere [55,62]. In brief, the PEGylation reaction was aliquoted at varying time intervals as indicated in the figures, and added to freshly prepared fluorescamine solution (0.05% in DMSO), and the resulting mixture was added to PBS. Fluorescence readings were performed using a Jasco FP–6300 spectrofluorometer (Jasco Inc, USA) with excitation at 390 nm and emission at 475 nm. The readings were corrected for their signal contributions with a blank sample. Further kinetic analysis was performed by C18-RP-HPLC and SDS-PAGE.

### Trypsin digestion of the PEGylated human amylin products

50 μg of the purified monoPEGylated human amylin product was subjected to trypsin proteolysis as follow. The PEGylated amylin was treated with 10 mM dithiothreitol in the presence of 25 mM ammonium bicarbonate for 30 min at 60°C, followed by blockage of the free cystein with iodoacetamide (55 mM in 25 mM ammonium bicarbonate for 30 min at 25°C protected form light). Trypsin was added (final concentration 5 μg/mL) and the digestion proceeded for 18 h at 37°C. The proteolysis was quenched by adding 10% volume of TFA 5% in CH_3_CN 50% and incubation at 37°C for 90 min. The solvent was removed by vacuum and the digested sample was subjected to MALDI-ToF-MS as described bellow. The expected digest products were calculated with http://web.expasy.org/peptide_mass/.

### Matrix-assisted laser desorption and ionization-time-of-flight mass spectrometry (MALDI-ToF-MS)

MALDI-ToF-MS was performed as a service provided by the CEMBIO-UFRJ. Data were acquired in an Autoflex Speed spectrometer (Bruker, USA) in positive linear mode and using sinapinic acid (10 mg/mL) as matrix. The samples were added of equal volumes of 50% acetonitrile in water containing 0.1% trifluoroacetic acid (TFA) and proceed for MALDI-ToF-MS analysis. The MS spectra were analyzed using mMass [63].

### Human RAMP3 Extracellular Topological Domain

The human receptor-activity modifying protein 3 Extracellular Topological Domain (RAMP3-ETD; residues 28–117; UNIPROT identifier O60896; Sequence in **S1 File**) was obtained by heterologous production in *E*. *coli* and further purified from inclusion bodies. The synthetic DNA sequence encoding the RAMP3-ETD of RAMP3 was cloned into bacterial protein expression vector pET28b (Novagen/EMD Millipore) from the synthetic gene using NdeI and EcoRI. The sequence and identity of the gene in the pET28b-RAMP3 construct was confirmed with DNA sequencing. The gene synthesis, cloning and sequencing services were provided by GenScript Corporation (Piscataway, NJ, USA).

RAMP3 was produced and purified as described previously for the homologous protein RAMP2 [56]. The RAMP3-ETD was produced as a fusion protein containing an N-terminal histidine tag and a thrombin protease cleavage site, by heterologous expression in *E*. *coli* BL21DE3pLys (1 mM IPTG, 37°C, 3 h). RAMP3-ETD was purified from inclusion bodies by denaturation with 6 M guanidinium chloride and then subjected to affinity chromatography in IMAC Sepharose FastFlow (GE Healthcare, Brazil). The purified RAMP3-ETD was refolded by rapid dilution into cold buffer 50 mM sodium phosphate buffer (pH 8.3) containing 1 M arginine hydrochloride, 5 mM reduced glutathione, 1 mM oxidized glutathione, and 1 mM EDTA and left stand for 24 h at 4°C. After refolding, RAMP3-ETD was dialyzed, concentrated in AMICON (Millipore, Brazil), and stored in 20 mM sodium phosphate buffer and 500 mM NaCl, pH 7.0, in liquid nitrogen until use. Purity was over 95% as judged by SDS-PAGE, routine size-exclusion chomatography and MALDI-ToF-MS. The purified RAMP3 was quantified by absorbance at 280 nm, using 21,345 M^-1^cm^−1^ as extinction coefficient.

### Human CTR1 Extracellular Topological Domain

The DNA sequence encoding the extracellular topological domain (ETD) of the human calcitonin receptor, isoform 1 (CTR1; residues 43–168, UNIPROT identifier P30988-1; MW 18062.2 Da; Sequence in **S1 File**) was synthesized *in vitro* and cloned into bacterial protein expression vector pET28b (Novagen/EMD Millipore) by GenScript Corporation (Piscataway, NJ, USA) using NdeI and EcoRI. The construct sequence and identity of the DNA gene in the pET28b-CTR1 construct was confirmed with DNA sequencing by GenScript using the T7 promoter/terminator primers.

The CTR1 was produced as a fusion protein containing an N-terminal histidine tag, a thrombin and a TEV protease cleavage sites. The protein was expressed in *E*. *coli* BL21DE3pLys (1 mM IPTG, 37°C, 3 h). The CTR1-ETD was purified from inclusion bodies after solubilization with 6 M guanidinium chloride and subjected to chromatographic purification by using the IMAC Sepharose FastFlow (GE Healthcare, Brazil) and refolded by rapid dilution into cold buffer 100 mM Na_2_CO_3_ (pH 10.5), 15 mM Na_2_HPO_4_, 228 mM NaCl, 5 mM reduced glutathione and 1 mM oxidized glutathione and left stand for 24 h at 4°C. The refolded CTR1-ETD was then dialyzed, concentrated and stored in 100 mM Na_2_CO_3_ pH 9.0, 15 mM Na_2_HPO_4_, 228 mM NaCl and 1 mM Na_2_H_2_.EDTA in liquid nitrogen until use. Purity was over 95% as judged by SDS-PAGE, routine size-exclusion chomatography and MALDI-ToF-MS. The purified CTR1 was quantified by absorbance at 280 nm, using 48,735 M^-1^cm^−1^ as extinction coefficient.

### Isothermal binding assays

PEGylated human amylin binding to the molecular partners CTR1 and RAMP3 and self-assembly (interaction with free murine amylin instead of free human amylin due to aggregation issues with the later) was assessed by changes in anisotropy fluorescence of the fluorescein-labeled proteins as follow. The proteins (CTR, RAMP and murine amylin) were labeled with fluorescein isothiocyanate (FITC; Sigma-Aldrich; 0.2 mM final concentration, from stock in DMSO at 5 mM freshly prepared) in 20 mM Na_2_HPO_4_, 300 mM NaCl, pH 7.0, for 60 min at 25°C protected from light. The reaction was quenched by adding an excess of Tris-HCl. The free fluorescein was removed by chromatography with a Sephadex G25 desalting column (GE Healthcare, Brazil). The labeling efficiency, final yield and protein quantification were calculated as described elsewhere [56]. The labeling efficiency was below 1 mol FITC:1 mol protein for all labeling procedures. Labeled proteins were stored until use protected from light at -20°C for no longer than 3 months.

Ligands (unlabeled murine amylin and PEGylated human amylin) binding to fluorescein-labeled CTR, RAMP and murine amylin were assayed at 25°C in PBS by monitoring the changes in the fluorescence anisotropy of the labeled proteins (50 nM) as a function of total ligand concentration. The fluorescence measurements were conducted in a Spectramax M5 (Molecular Devices) microplate reader, with excitation and emission set at 480 nm and 520 nm respectively, with cut-off filter 515 nm. The results represent the mean and the standard error of the mean (SEM) of five measurements. Binding data analysis was performed with SigmaPlot 12.5 (Systat Software, Inc., CA, USA).

### cAMP production in MCF–7 cells following stimulation with amylin and PEGylated amylin

The production of cAMP in response to stimulation with non-conjugated or PEGylated human amylin was acessed in MCF–7 cell lines. The assay was conducted by the CRO Eurofins | Cerep Panlabs, using the cAMP HiRange assay kit (Cisbio, 62AM6PE). According to the company, the assay was performed in 96 well half area plates, in a total reaction volume of 20 μl/well, with a total of 3.000 cells in suspension (after optimization for optimal seeding density and format: adherent or suspension), freshly thawed. Cells were incubated at 37°C in a humidified atmosphere with 5% (v/v) CO_2_. For stimulation, cells were incubated for 30 minutes at 37°C in HBSS supplemented with 20 mM HEPES (pH 7.4) and 500 μM IBMX (phosphodiesterase inhibitor) in the presence of non-conjugated human amylin (purchased from Bachem by the Eurofins) vs. PEGylated human amylin. Following the incubation, cells were lysed and the cAMP quantified by using the cAMP HiRange assay kit (Cisbio, 62AM6PE) according to the manufacturer instructions. In brief, the fluorescence probes, both acceptor (D2-labeled cAMP) and donor (anti-cAMP antibody labeled with europium cryptate) were added. cAMP produced by the cells following stimulation with nonconjugated or PEGylated human amylin competes with the labeled cAMP, thus diminishing the FRET (Förster resonance energy transfer) between D2 and cryptate on the labeled cAMP and antibody, respectively. After 60 minutes incubation at room temperature, fluorescence was measured (excitation at 337 nm, emission at 620 vs. 665 nm), and the cAMP concentration was determined by dividing the signal at 665 nm by that at 620 nm and comparing with the cAMP calibration curve. Buffer was used as negative control and the results were corrected accordingly. Results are expressed as mean ± standard error (n = 2) of the resulting cAMP concentrations in nM. EC_50_ values for the agonist-induced cAMP production was calculated by adjusting data with a logist 4 parameter function (with SigmaPlot, Jandel Sci). Data were obtained from a third party, i.e., the authors did not generate the primary dataset themselves

### Amylin aggregation assay

The aggregation kinetic of free of PEGylated human amylin (10 mM buffer, 20 μM ThT, 50 μM peptide = 0.2 mg/mL, with 2% DMSO final concentration,) was performed in either continuous measurement in a multiwell plate (Corning Costar black plate; in a SpectraMax M5 spectrofluorimeter) or in separated samples (by using a Jasco FP6300 spectrofluorimeter). In this case, at the indicated time 160 μL sample were mixed with 40 μL ThT 100 μM. In both cases the thioflavin T fluorescence was measured by setting excitation at 450 nm and emission at 482 nm. The presence of residual (2%) DMSO shows no significant effect on human amylin aggregation as reported elsewhere [64] and in our own control experiments performed with varying concentration of DMSO (**S1 Fig)**, demonstrated no significant contribution up to about 6% DMSO. The aggregation curves were fitted using a 4 parameter logistic function and the t_1/2_, elongation rate and lag time were estimated as described elsewhere [65].

### Morphologic analysis by transmission electron microscopy (TEM)

Samples from aggregation kinetic assay were applied to 300 mesh, Formvar-carbon-coated Cu grids (Electron Microscopy Science, USA), followed by negative stain with 2% uranyl acetate, pH 4.8, for 30–60 s. Stained samples were observed in a transmission electron microscope (Tecnai, FEI) operating at 120 kV.

### Pharmacological evaluation of PEGylated amylin

The pharmacological evaluation of amylin products was performed as described below.

#### Pharmacokinetics

Swiss male mice (8 weeks old; n = 4 per group) were administrated of free or monoPEGylated human amylin (both at 400 μg peptide / kg body weight) by subcutaneous route. At the indicated time interval, blood was collected with glass capillary tubes from retro-orbital access and the serum was separated. The concentration of amylin in the plasma was evaluated by ELISA (Merck Millipore, cat # EZHA-52K) by following the manufacturer protocol. The sensitivity of the ELISA assay kit for the monoPEGylated human amylin product was similar to the non-conjugated human amylin as inferred from control analytical curves. In case needed, the mice plasma samples were diluted accordingly in order to achieve the desired concentration range of the ELISA kit. Glucagon was evaluated from the same serum samples, by using an ELISA kit (Merck Millipore, Cat # EZGLU-30K) according to instructions of the manufacturer.

#### Pharmacodynamic evaluation

The pharmacological effect of human amylin over mice glycemia was conducted as described previously [4,55]. In brief, eight-week-old Swiss male mice (25 g ± 1 g) were divided into three groups as follows: control (receiving regular insulin at 0.3 IU/kg body weight; n = 5), amylin (receiving 400 μg murine amylin/kg body weight plus regular insulin at 0.3 IU/kg body weight; n = 5) and PEGylated amylin (receiving 400 μg PEGylated human amylin/kg body weight plus regular insulin at 0.3 IU/kg body weight; n = 5). The animals were housed in a temperature-controlled room with a light-dark cycle of 12 h. Water and food were available *ad libitum* and suspended 6 h before the experiments, and kept fasting throughout the experiments. The groups received 100 μL of the formulations by subcutaneous (s.c.) route using a standard 29 gauge needle (BD^TM^).The glycemia was monitored by whole blood samples obtained from the tail tips of conscious, unrestrained mice using pre-calibrated point-of-care glucometers (Accu-Chek Active, Roche Diagnostics, Germany), as described elsewhere [55]. The animals were decapitated at the end of the experimentation. This protocol was approved by the Institutional Bioethics Committee on Animal Care and Experimentation at UFRJ **(# FARMACIA05/2012)**. Statistical analysis was performed using one way analysis of variance (ANOVA) with Bonferroni test as post hoc analysis by using GraphPad Prism ver 5.01 (GraphPad Software). A p-value of <0.05 was considered to be significant (**S1 Table**).

## Results

### Development of PEGylated human amylin

Amylin, both human and from other organisms, display very limited solubility in aqueous milieu and a propensity to form amyloid aggregates [45–47,66–68]. Aiming to achieve a physico-chemical stable form of the human amylin which would still bear its functional activity, we designed the conjugation of the human amylin with the hydrophilic PEG moiety. The conjugation of PEG with proteins is typically conducted in aqueous solvent [69]. However, due to the incompatibility of aqueous milieu with the human amylin we opted for using in organic solvent instead [70]. DMSO was the solvent of choice given the solubility of both PEG and amylin, the reduced volatility of this solvent and its compatibility with further chromatographic purification steps.

**Fig 2:**
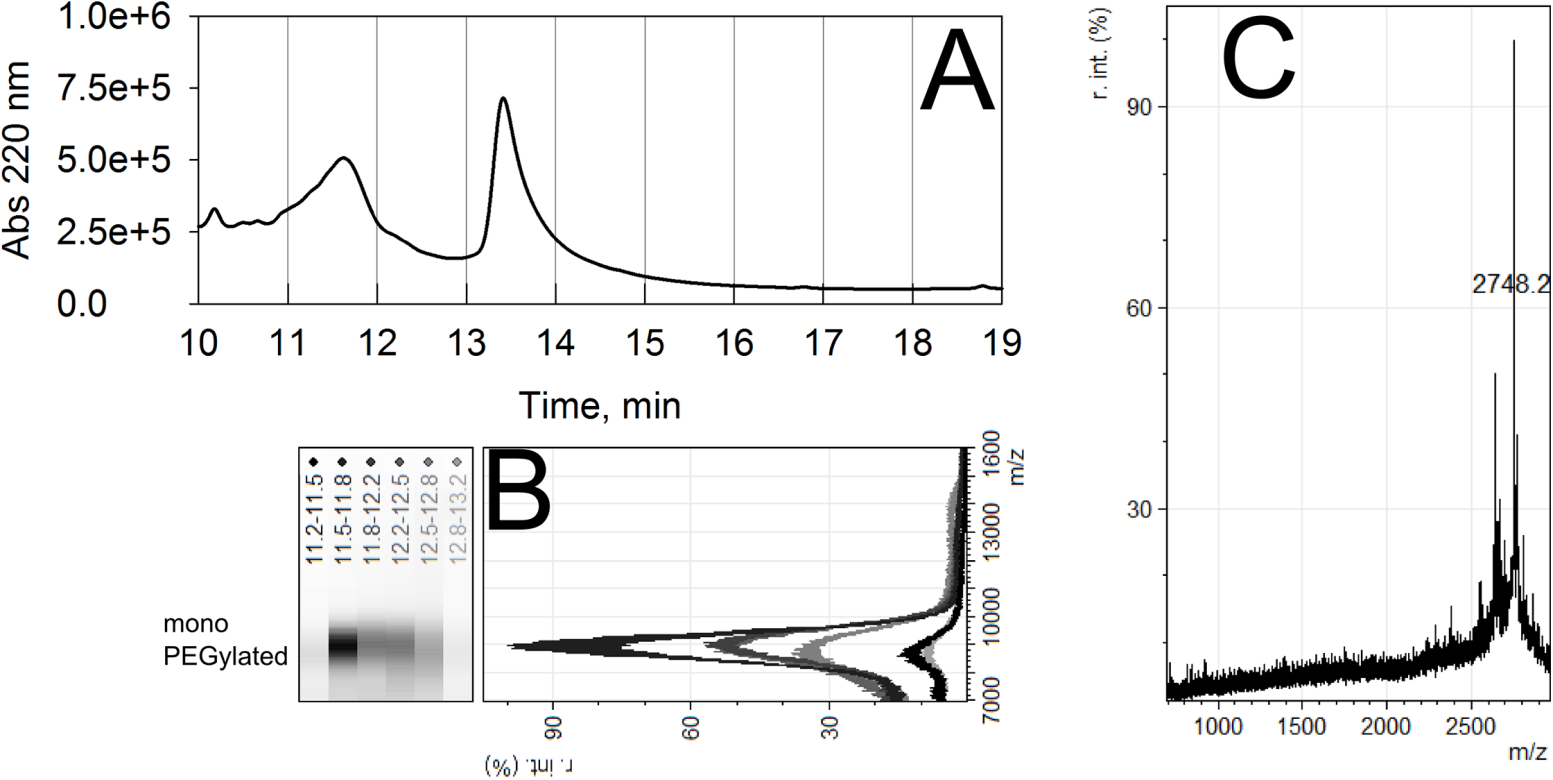
Purification and characterization of monoPEGylated human amylin. **a)** MonoPEGylated human amylin was synthesized in organic solvent by conjugation human amylin (5 mg/mL) with mPEGsc5k (5:1 molar excess) and purified by C18-RP-HPLC with a 30–70% CH_3_CN gradient (in the presence of 0.1% TFA) up to 20 min. **b)** MALDI-ToF-MS of the fractions from the purification step showing the mono PEGylated human amylin corresponding to the peak comprising the elution time 11 min–13 min. The panel is rotated in order to align the PAGE-like display of the mass spectra with the respective original fractions in the chromatogram (upper panel, A). **c)** Trypsin-digestion of PEGylated human amylin. Purified monoPEGylated human amylin was subjected to trypsin digestion and submitted to MALDI-ToF-MS for identification of the products. The 2748 m/z ion coincides with the expected monoisotopic mass for the sodium adduct of the non-modified (non-PEGylated) human amylin 12–37 fragment (amylin 12–37, amide at C-terminus; monoisotopic mass 2,725.3 Da)

We thus decided to use a PEGylation condition for the human amylin in consonance with the reaction conditions used for the murine amylin [56], i.e., 5 mg/mL amylin, 25°C and 5:1 molar excess mPEGsc5k. However, the PEGylation reaction at these conditions resulted in limited reaction up to 4 h, regardless of the molar excess of mPEGsc5k (**S2 Fig**). Previous observation from our group noticed that some chemically synthesized peptide batches display varying residual acidity, which impairs the conjugation reaction. Thus we decided to use a limited amount of a mild buffer (PBS) in the PEGylation reaction, which has been satisfactorily used for PEGylation of murine amylin [56], allowing the conjugation reaction. The purification of the PEGylation product resulted in a well-resolved peak in a C18-RP-HPLC purification procedure (**Fig 2A**), with high purity monoPEGylated human amylin (**Fig 2B)**. In over 10 batches produced in our lab in varying scales (from 2.5 to 5.0 mg human amylin), we have obtained a typical yield of about 30–40% (expressed as mass of the peptide moiety recovered) monoPEGylated human amylin.

The identification of the conjugation site was conducted by proteolysis of the monoPEGylated human amylin with trypsin followed by MALDI-ToF-MS. Trypsin cleaves after Lys_1_ and Arg_11_, which might result in up to two amylin peptide fragments and derivatives: amylin 2–11 (monoisotopic mass 1,067.4 Da), the 2 carbamidomethyl-cysteines amylin products 2–11 (from the acylation with iodoacetamide; monoisotopic mass 1,181.5 Da) and amylin 12–37 (amide at C-terminus; monoisotopic mass 2,725.3 Da). We have found the ion 2,748.2 m/z, which is compatible with the unmodified amylin 12–37 sodium adduct. No other ion was found at lower m/z, suggesting that PEGylation occurred in the initial, N-terminal region of amylin, which comprises the free amino groups from the Lys_1_ targeted for PEGylation. These data corroborate the decrease in availability of free primary amines in the course of the PEGylation reaction as probed by the specific fluorescent probe fluorescamine (**S3 Fig**), indicative of the preference for amine by the PEG-NHS conjugation agent.

### Stability of PEGylated human amylin

**Fig 3:**
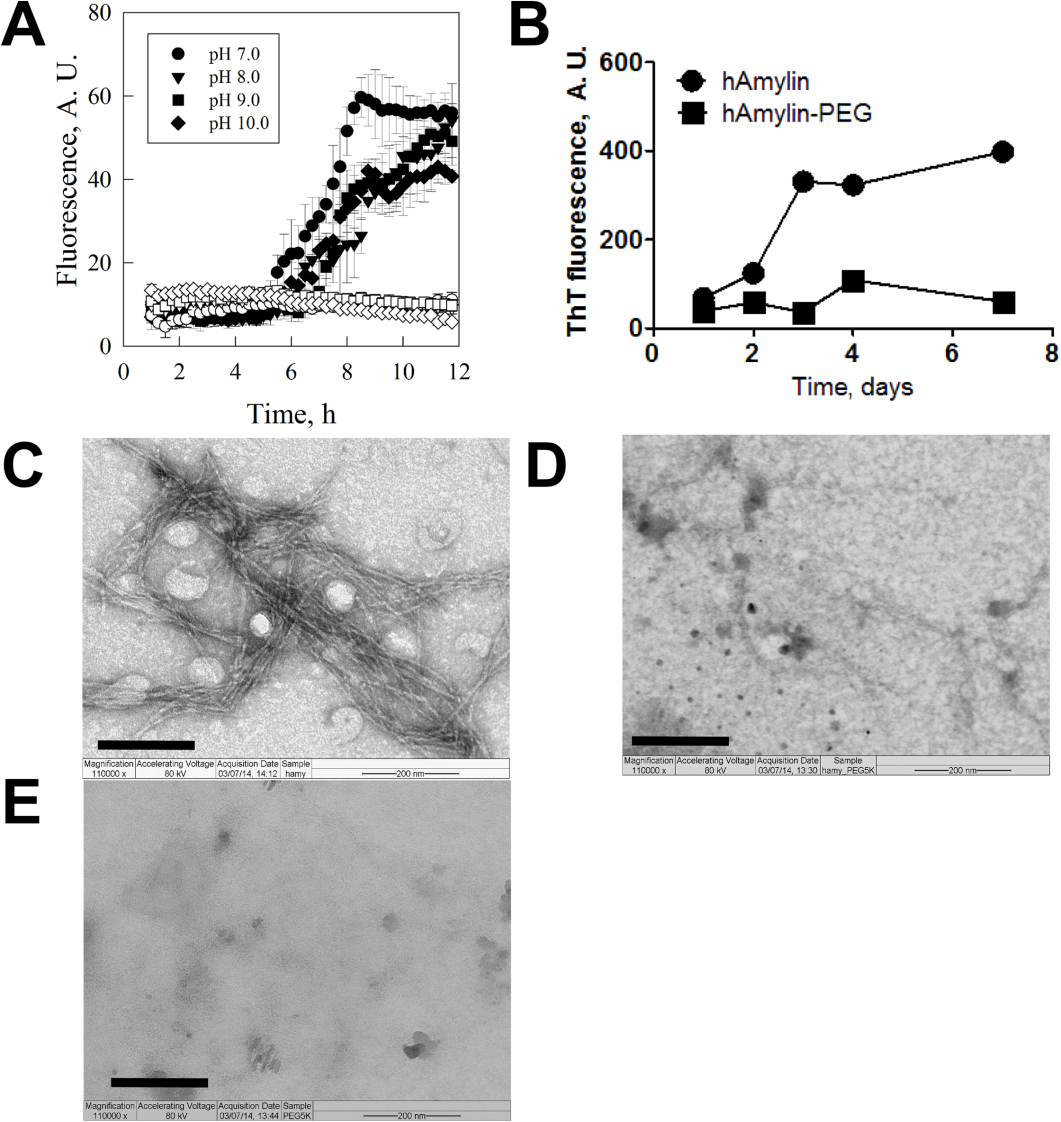
Physical stability of the free and the PEGylated human amylin. Human amylin was subjected to aggregation at 25°C and monitored for fibril formation by ThT fluorescence **(a)** for up to 12h at varying pH (closed symbols, free non-conjugated human amylin; open symbols, PEGylated human amylin) and **(b)** for up to 7 days in PBS pH 7.4, and the products of these aggregation kinetic isotherms were further evaluated by transmission electron microscopy (TEM) as follow: **(c)** free human amylin; **(d)** PEGylated human amylin; **(e)** free PEG. Scale bar = 200 nm. The raw data can be found in **S2 File**.

The physical stability of monoPEGylated human amylin product was evaluated by measuring its propensity for amyloid aggregation in comparison to the non-conjugated human amylin. In the aggregation assay we used the specific dye ThT which enhances its fluorescence upon binding to amyloid fibrils. The assays conducted with the non-conjugated human amylin showed a lag phase of about 5 h followed by the progressive increase in the fluorescence, reaching a plateau at about 8 to 10 h (**Fig 3A**). In this same time windows we did not observe changes in fluorescence in the assay conducted with the PEGylated human amylin product. We have further extended the aggregation assay following for up to 7 days. The aggregation isotherm of human amylin is further increased after the fast, first phase of amylin aggregation (which took place within the 12 h window). The second aggregation phase reached a stable plateau after 3 days, while only minor changes in ThT fluorescence was observed for the PEGylated human amylin (**Fig 3B**). The morphologic analysis of the resulting material by TEM shows typical amyloid fibrils for the aggregation assay performed with free human amylin (**Fig 3C)**, while no fibrils were observed in the aggregation performed with PEGylated human amylin (**Fig 3D**) or in the control assay performed with free mPEGsc (**Fig 3E).**

### Amylin self-assembly and interaction with receptor

**Fig 4:**
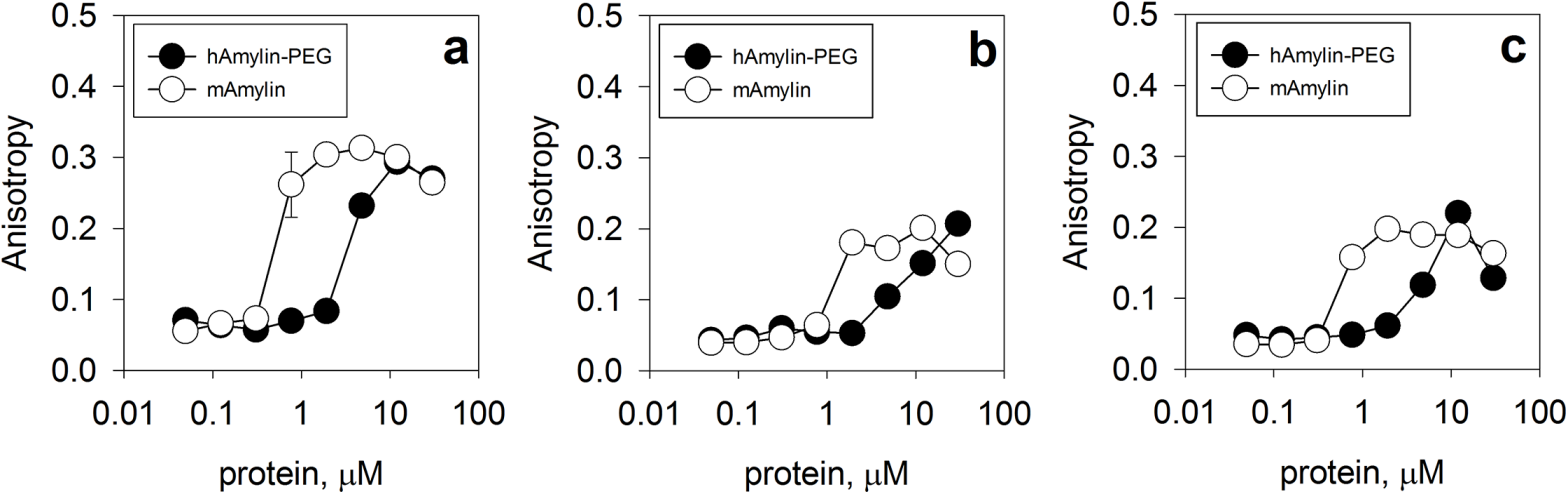
Amylin self-assembly and interaction with co-receptors. Free murine amylin or PEGylated human amylin were assayed for binding with (**A**) murine amylin, (**B**) CTR–1 and (**C**) RAMP–3 by fluorescence anisotropy of fluorescein labeled molecular partners. Assays were conducted in PBS, pH 7.4, 25°C, in the presence of 50 nM FITC-labelled proteins (amylin, CTR–1 or RAMP–3). Ex 480 nm, Em 520 nm, filter 515 nm. The raw data can be found in **S2 File**.

In order to further evaluate the function of the stable, monoPEGylated human amylin, we performed isothermal binding assays with the known molecular partners. For this purpose a direct comparison of the PEGylated product with the non-conjugated human amylin would not be possible due to its propensity for aggregation. However, the murine amylin is well known for its increased solubility and less-prone aggregation behavior [45,46,49], allowing its use in a pairwise comparison to PEGylated human amylin. Amylin, RAMP and CTR were labeled with fluorescein and binding with non-conjugated and with the PEGylated amylin was measured. Both free and PEGylated amylin are able to performing self-assembly with amylin (**Fig 4A**) and with the coreceptors CTR1 (**Fig 4B**) and RAMP3 (**Fig 4C**). The specificity of this binding assay has been previously reported elsewhere by evaluation with non-relevant ligands [56,71]. Despite the minor shift to higher concentration of protein is observed for PEGylated amylin, which might be due to steric hindrance due to the PEG moiety or further conformational changes in the PEGylated amylin, PEGylation did not abolish the assembly.

### Amylin activity in cell

**Fig 5:**
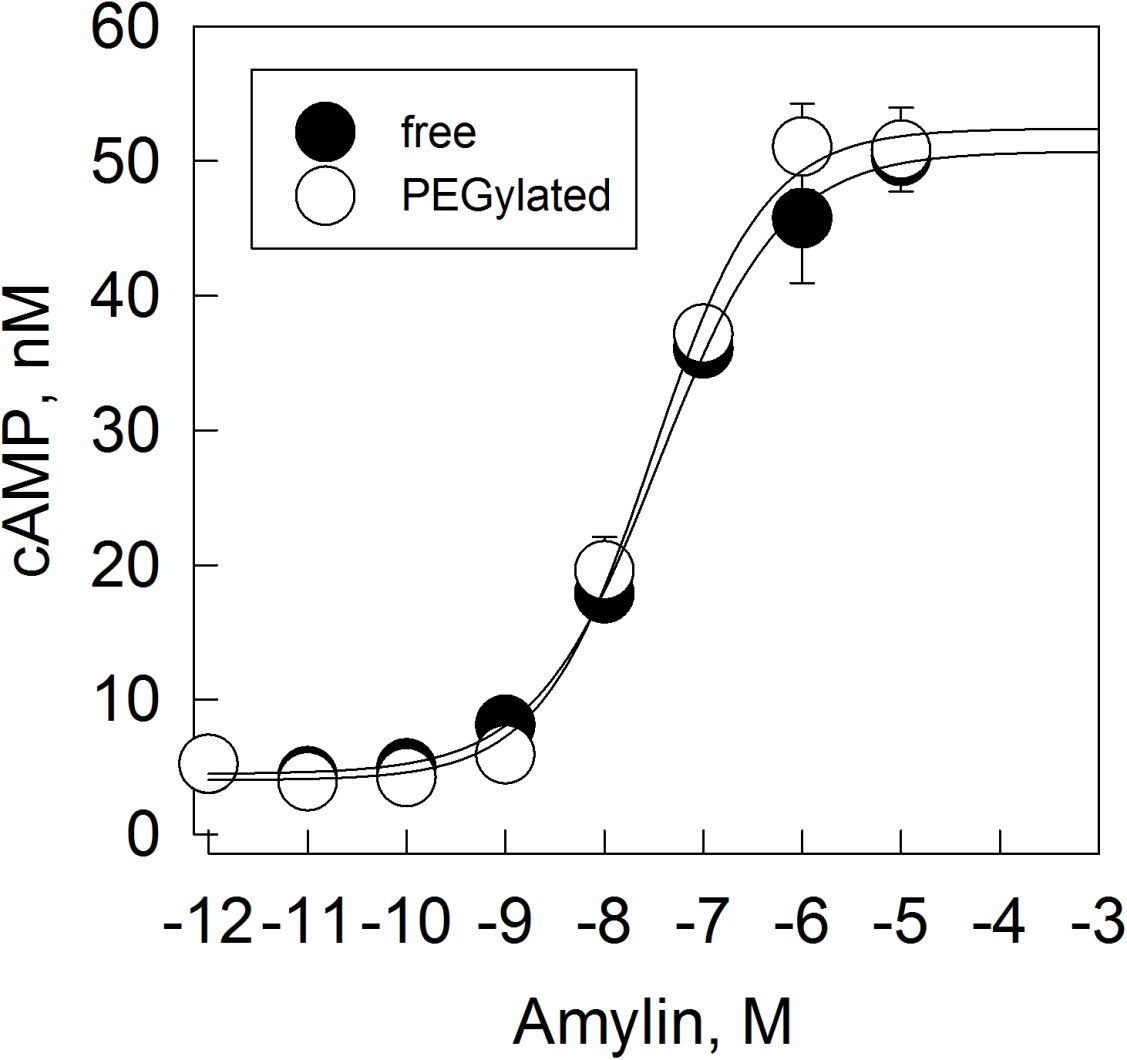
Amylin-stimulated generation of cAMP in MCF–7 cells. Non-conjugated or PEGylated amylin were assayed for activity in cell by means of stimulation of production of cAMP in MCF–7 cell line. Lines represent best adjust of experimental data with logistic function. Human amylin EC50 = 35.2 ± 7.5 nM, PEGylated human amylin EC50 = 30.8 ± 6.7 nM. The raw data can be found in **S2 File**.

To further characterize the effect of the PEG moiety on recognition by the co-receptors, we evaluated the response to PEGylated human amylin in cells. MCF–7 cells endogenously express human amylin receptor CTR1 and CTR2 [72,73]. Stimulation of the receptor with amylin results in the production of cAMP (**Fig 5**). The free, non-conjugated human amylin resulted in an EC50 of 35.2 ± 7.5 nM (r2 = 0.990), and the PEGylated human amylin resulted in a EC50 of 30.8 ± 6.7 nM (r = 0.988). These data indicates the similarity of response of both non-conjugated and the PEGylated human amylin in MCF–7 cells.

### Pharmacological evaluation of PEGylated human amylin

We evaluated the pharmacological activity of the PEGylated human amylin by three assays: the pharmacokinetics, the modulation of glycemia and the modulation of glucagon.

**Fig 6:**
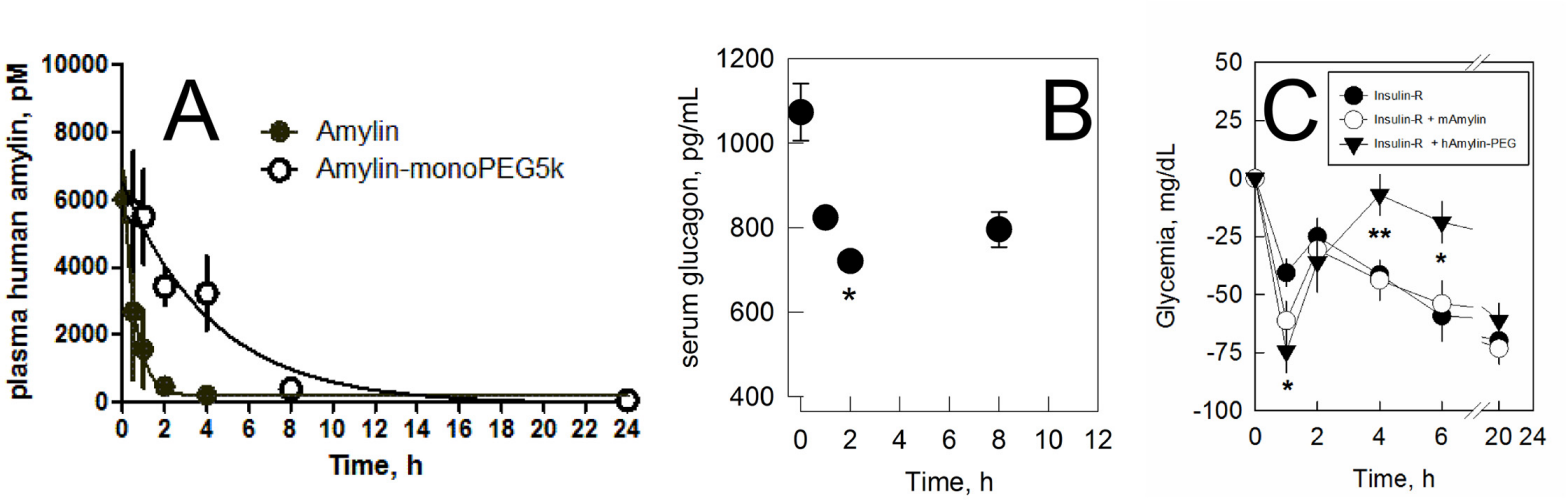
Pharmacologic evaluation of PEGylated human amylin. **a)** Pharmacokinetics. Non-conjugated and PEGylated human amylin were injected to two separated groups of swiss male mice by subcutaneous administration and the decay in plasma concentration was evaluated. Continuous lines are best fitting of single-exponential decay function to data. **b)** Modulation of glucagon. Swiss male mice were administered with 10 μg PEGylated human amylin and the serum glucagon was monitored over time. * P<0.05. **c)** Modulation of glycemia. Swiss male mice (8 weeks old; fasting for 6 h before intervention and throughout the experiment) received by subcutaneous injection regular insulin (0.3 IU/kg body weight) alone (closed circles; n = 5) or in combination with free murine amylin (open circles; 400 μg/kg body weight; n = 5) or PEGylated human amylin (closed inverted triangles; 400 μg/kg body weight, expressed as peptide fraction; n = 5) and glycemia was monitored in the tail tip of free, conscientious mice. The activity of PEGylated human amylin showed significant difference from control (Insulin-R): * P<0.05, **P<0.01. The raw data can be found in **S2 File**.

The free and the PEGylated human amylin were injected by subcutaneous route in separated groups of swiss male mice. At given time intervals, the bioavailability of amylin in serum was evaluated by ELISA. We observed a typical PK curve for the free amylin, with a half-time of 23 min (**Fig 6A**). The PEGylated amylin showed a slower decrease in disappearance rate from plasma, resulting in a half-life of 179 min, 7.8 times greater than the unmodified amylin.

PEGylated amylin was further evaluated for its ability in modulation of glucagon. A progressive decrease in serum glucagon level is observed following the subcutaneous administration of PEGylated human amylin, reaching a minimum at 2 h with subsequent increase in glucagon level (**Fig 6B)**, demonstrating the efficacy of the conjugated hormone.

We have further evaluated the effect of PEGylated human amylin over the glycemia. Both insulin and non-conjugated or PEGylated human amylin were administrated by subcutaneous route in swiss male mice and the changes in glycemia was monitored over time. We observed a complex behavior, with a decrease in glycemia with maximum at about 1 h followed by a recovery in glycemia, which was similar in the control groups (only insulin or insulin plus non-conjugated amylin) but sustained for 6 h in the group receiving PEGylated human amylin (**Fig 6C)**. Collectively, these data indicate the sustained acting behavior of the PEGylated human amylin *in vivo*.

The exact mechanism for the observed pharmacological response is not clear for us, and deserves future in deep investigations. In fact, we observe a dual behavior, the first phase while under the action of insulin (which show no longer significance after 2h compared with regular insulin + free amylin) and a second, long-lasting effect, after the effect of the regular insulin. Such behavior suggest that the PEGylated human amylin shows a pharmacological peak, which is not the ideal for sustained-action pharmaceuticals thought not surprisingly since this substance was not designed to behave as a depot upon subcutaneous administration. However, the PD behavior shown here should not be understood as an idealized therapeutic scheme. In fact, the eventual progress of the development of a PEGylated (either 5kDa or any other polymeric moiety) amylin or an amylinomimetic compound as a pharmaceutical agent would deserve further pharmacological evaluation under a varying of dissimilar schemes, such as its use with fast acting, regular or slow-acting insulin analogues and the evaluation in animal models of T1DM and T2DM

## Discussion

Since the introduction of insulin as the first therapeutic agent in the treatment of diabetes [74] there has been no further advances in the introduction of novel products addressing the T1DM [39], in particular targeting the fasting state, which is highly governed by glucagon and results in high glycemic excursions in the patient [75]. The landmark DCCT study in tight control of diabetes (https://clinicaltrials.gov/ct2/show/NCT00360815) have shown that a well controlled diabetes result in the reduction of the risks for diabetes complications [76], although the high intensive therapy targeting lower glucose levels has been followed by the rise in numbers of hypoglycemic events [77,78], urging for the need of additional therapeutics with both cardiovascular and hypoglycemic safety assured. In this context, a better control of the post-prandial metabolic homeostasis is highly desirable, which should include not only basal insulin but also basal amylin, the ultimate hormone in the control of glucagon.

Human amylin is known for its low solubility in aqueous milieu, prompting for the formation of well-documented oligomer-like and amyloid fiber structures. These characteristics have hampered the development of therapeutics based on homologous replacement with human amylin, which resulted in the development of a triple-mutant amylin analogue which has been made available commercially since 2005.

Despite its notable physiological importance for homeostatic control, the amylinomimetic composition has not impacted extensively the therapy in diabetes [79], although recommended by the American Diabetes Association for both T1DM [39] and T2DM [33]). The observation of physical interaction between amylin and insulin has been extensively reported [22–26,52,80], resulted in the recommendation for separated administration [81], and thus discouraging patient for the adjunctive therapy with amylinomimetic compound (due to an additional injection along with insulin), despite the benefits for the glycemic control.

One additional limitation for the use of the currently available amylinomimetic compound is the lack of ability for mimicking the basal levels of amylin in the fasting state. Amylin has a short half-life (t_1/2_) of approximately 15–20 minutes [49] (**Fig 6A)** and thus a sustained release of amylin of an increasing in its t_1/2_ would serve as potential strategies for the restoration of basal hormone levels. We have previously demonstrated that a controlled and sustained release of human amylin in a bioactive form could be achieved from polymeric particles as a depot formulation [55]. Additionally, we have shown that using the strategy of bioconjugation of murine amylin with PEG could provide extension of its effect *in vivo* [56,71].

We have shown here that human amylin can be conjugated with methoxyl PEG carbonate, resulting in the monoPEGylated hormone. The optimization of the synthesis and purification resulted in the monoPEGylated human amylin product in high final yield and purity. The monoPEGylated human amylin obtained here demonstrated to behave as a stable, water soluble and bioactive hormone with about 8 times longer half-life *in vivo* compared to the unmodified hormone. PEGylation of human amylin could be achieved by using a molar ratio of 5 mol PEG:1 mol amylin, resulting in about 30–40% of monoPEGylated amylin after 2 h reaction in DMSO, which is both a satisfactory solvent for dissolution of amylin peptides and compatible with further chromatographic purification strategies.

## Conclusions

Formulation and the design of pharmaceutical compositions with biologicals is particularly challenging, including the propensity for aggregation into oligomers and amyloid fibril, both for amylin and two other pancreatic hormones: insulin [82] and glucagon [83]. Strategies in formulating proteins and peptides must address challenging issues including solubility, long term chemical and physical stability, biocompatibility, bioavailability, immunogenicity (even for synthetic peptides / proteins due to conformational, polymorphic variability) and toxicity. For over 25 years the availability of homologous human amylin therapeutics has been halted due to the limited aqueous solubility of the peptide [45,80]. We show here that the present approach of conjugation of a single PEG moiety to the N-terminus of the human amylin peptide chain confers a prominent increase in solubility (to over 10 mg/mL, calculated in the peptide moiety basis), conferring enhanced physical stability against aggregation while providing equivalent receptor response and extended *in vivo* half-life and activity. We believe that the present conjugate might benefit the development of novel therapeutics addressing diabetes mellitus.

## Supporting Information

S1 Fig Effect of DMSO on the human amylin aggregation. Human amylin (50 μM) was incubated at 25°C in 10 mM Na_2_HPO_4_ pH 7.4 and 20 μM ThT and varying concentration of DMSO (as indicated in the legend) and the fluorescence was monitored (ex 440 nm, em 520 nm, filter cut-off 50% at 515 nm). The curves were adjusted with a logistic function and from the fitting parameters we calculated the B) t_1/2_ and lag time and C) elongation rate.
(TIF)

S2 Fig Kinetics of human amylin conjugation with mPEG5k in organic solvent in the absence of buffer. Human amylin (5mg/mL) was subjected to reaction with mPEGsc5k in DMSO at 25°C and at given time intervals aliquots were collected and conjugation products resolved in a 22% SDS-PAGE, and varying molar ratios with mPEG5k as follow: 2:1, 5:1, and 10:1. Lanes: Ladder, 0, 10 sec, 1 min, 10 min, 20 min, 30 min, 1h, 2h, 4h. Gels were stained with Coomassie Blue (for the detection of protein moiety, blue bands) followed by iodine staining (for the detection of PEG; brown bands). SDS-PAGE stained for protein (Coomassie, panels A, C and E) and PEG (Barium–Iodine, panels B, D and F) of the kinetics of human amylin (5 mg/mL) conjugation with mPEGscNHS-5k performed at varying PEG:amylin molar ratio. Reaction performed in a 2:1 molar ratio evidences incomplete reaction up to 4h (panel A and B). Increasing PEG:amylin concentration ratio to 5:1 and 10:1 results in increasing yield of pegylated amylin products, as observed by the decrease in the amount of remaining free amylin and the increasing amount of pegylated products. Reaction conducted for 4h resulted in formation of pegylated amylin products with high molecular order (slow migration in the SDS-PAGE; panels C and D). Reaction conducted up to 2h in either 5:1 (panels C and D) or 10:1 (panels E and F) molar ratio ensured a high consumption of the amylin (as indicated by the remaining free amylin) and prevention of extensive formation of higher molecular order pegylated amylin products.
(TIF)

S3 Fig PEGylation kinetic of human amylin in the absence of buffer monitored by fluorescamine. Human amylin (5 mg/mL) was incubated at 25°C in DMSO with varying amount of mPEGsc5k and the kinetics followed by quantifying the remaining primary amines with fluorescamine (10 μL reaction milieu + 200 μL fluorescamine 0.5 mg/mL in DMSO + 200 μL PBS, followed by immediate fluorescence reading). ***Inset*:** log scale.
(TIF)

S1 File The aminoacid sequence of the clones. a) Human CTR1 Extracellular Topological Domain (ETD) -pET28b. b) Human RAMP3 Extracellular Topological Domain (ETD)—pET28b.
(PDF)

S2 File Raw Data from Fig 3, Fig 4, Fig 5, Fig 6 and S1 Fig. (XLSX)

S1 Table Statistical Analysis of the Pharmacologic Evaluation–Glycemia. a) Two tailed p value. Insulin: hAmylin-PEG *versus* Insulin. b) Two tailed p value. Insulin:hAmylin-*PEG versus* Insulin:mAmylin. c) Two tailed p value. Insulin:hAmylin-PEG *versus Insulin*:hAmylin.
(PDF)

## Abbreviations

mPEG-SC: methoxylpolyethylene glycol succinimidyl carbonate
MALDI-ToF-MS: matrix-assisted laser desorption and ionization–time of flight–mass spectrometry
