# Supplementary figures and images for: Monoconjugation of Human Amylin with Methylpolyethyleneglycol
Source: PLoS One. 2015 Oct 8;10(10):e0138803. doi: 10.1371/journal.pone.0138803 (PMC4598023; doi:10.1371/journal.pone.0138803)

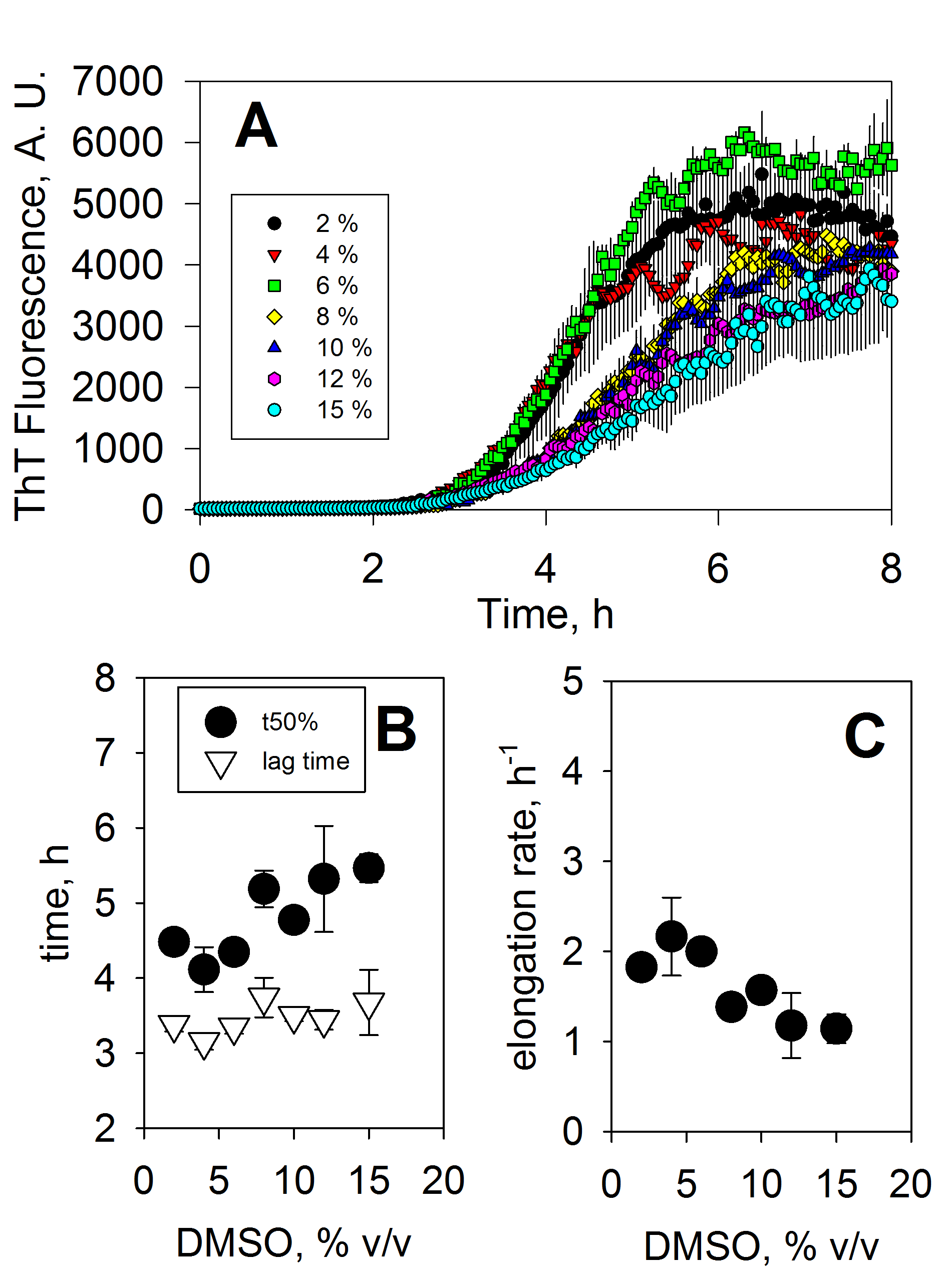

Supplement: S1 Fig — Human amylin (50 μM) was incubated at 25°C in 10 mM Na2HPO4 pH 7.4 and 20 μM ThT and varying concentration of DMSO (as indicated in the legend) and the fluorescence was monitored (ex 440 nm, em 520 nm, filter cut-off 50% at 515 nm). The curves were adjusted with a logistic function and from the fitting parameters we calculated the B) t1/2 and lag time and C) elongation rate. (TIF) [file pone.0138803.s001.tif]

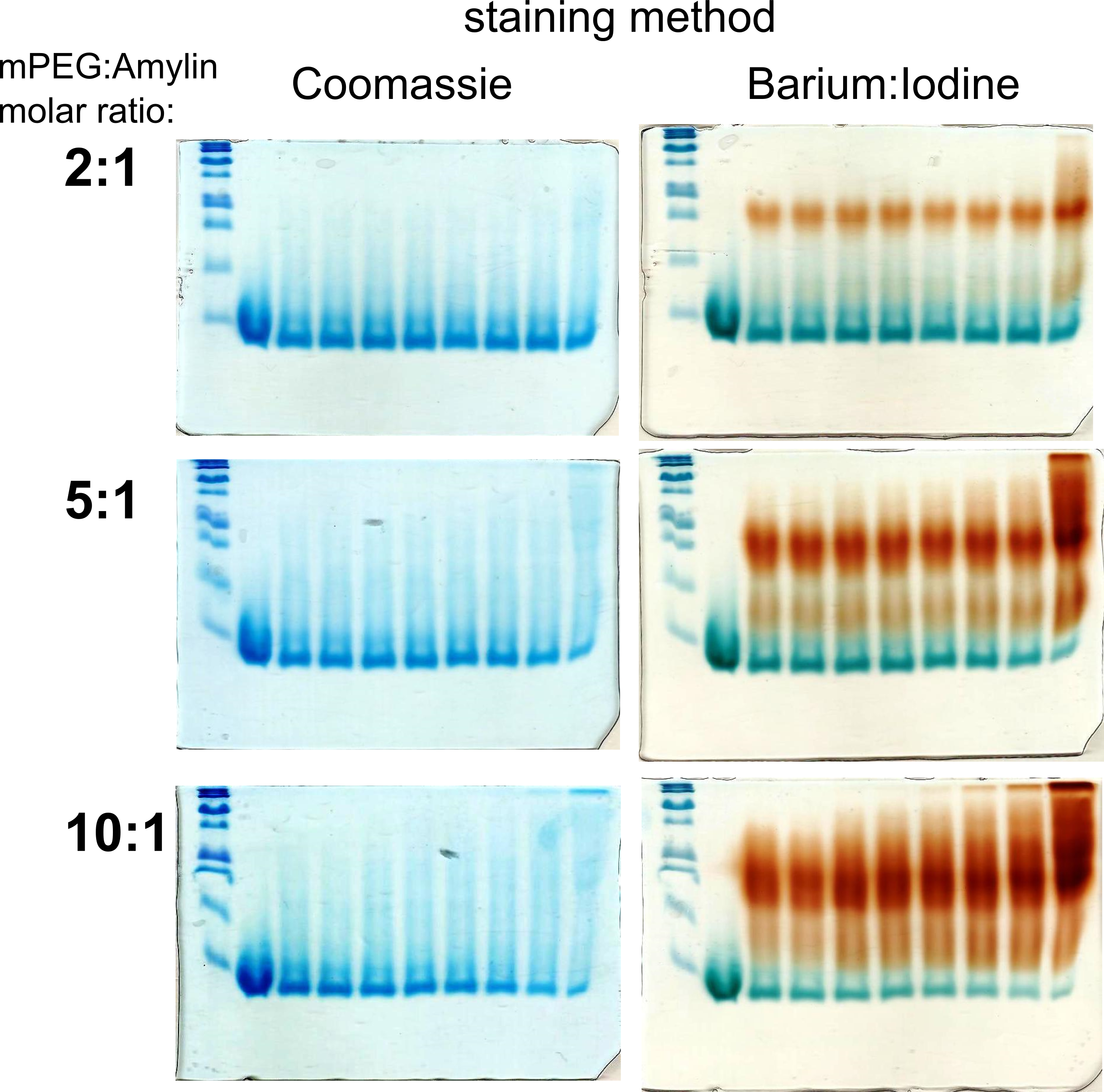

Supplement: S2 Fig — Human amylin (5mg/mL) was subjected to reaction with mPEGsc5k in DMSO at 25°C and at given time intervals aliquots were collected and conjugation products resolved in a 22% SDS-PAGE, and varying molar ratios with mPEG5k as follow: 2:1, 5:1, and 10:1. Lanes: Ladder, 0, 10 sec, 1 min, 10 min, 20 min, 30 min, 1h, 2h, 4h. Gels were stained with Coomassie Blue (for the detection of protein moiety, blue bands) followed by iodine staining (for the detection of PEG; brown bands). SDS-PAGE stained for protein (Coomassie, panels A, C and E) and PEG (Barium–Iodine, panels B, D and F) of the kinetics of human amylin (5 mg/mL) conjugation with mPEGscNHS-5k performed at varying PEG:amylin molar ratio. Reaction performed in a 2:1 molar ratio evidences incomplete reaction up to 4h (panel A and B). Increasing PEG:amylin concentration ratio to 5:1 and 10:1 results in increasing yield of pegylated amylin products, as observed by the decrease in the amount of remaining free amylin and the increasing amount of pegylated products. Reaction conducted for 4h resulted in formation of pegylated amylin products with high molecular order (slow migration in the SDS-PAGE; panels C and D). Reaction conducted up to 2h in either 5:1 (panels C and D) or 10:1 (panels E and F) molar ratio ensured a high consumption of the amylin (as indicated by the remaining free amylin) and prevention of extensive formation of higher molecular order pegylated amylin products. (TIF) [file pone.0138803.s002.tif]

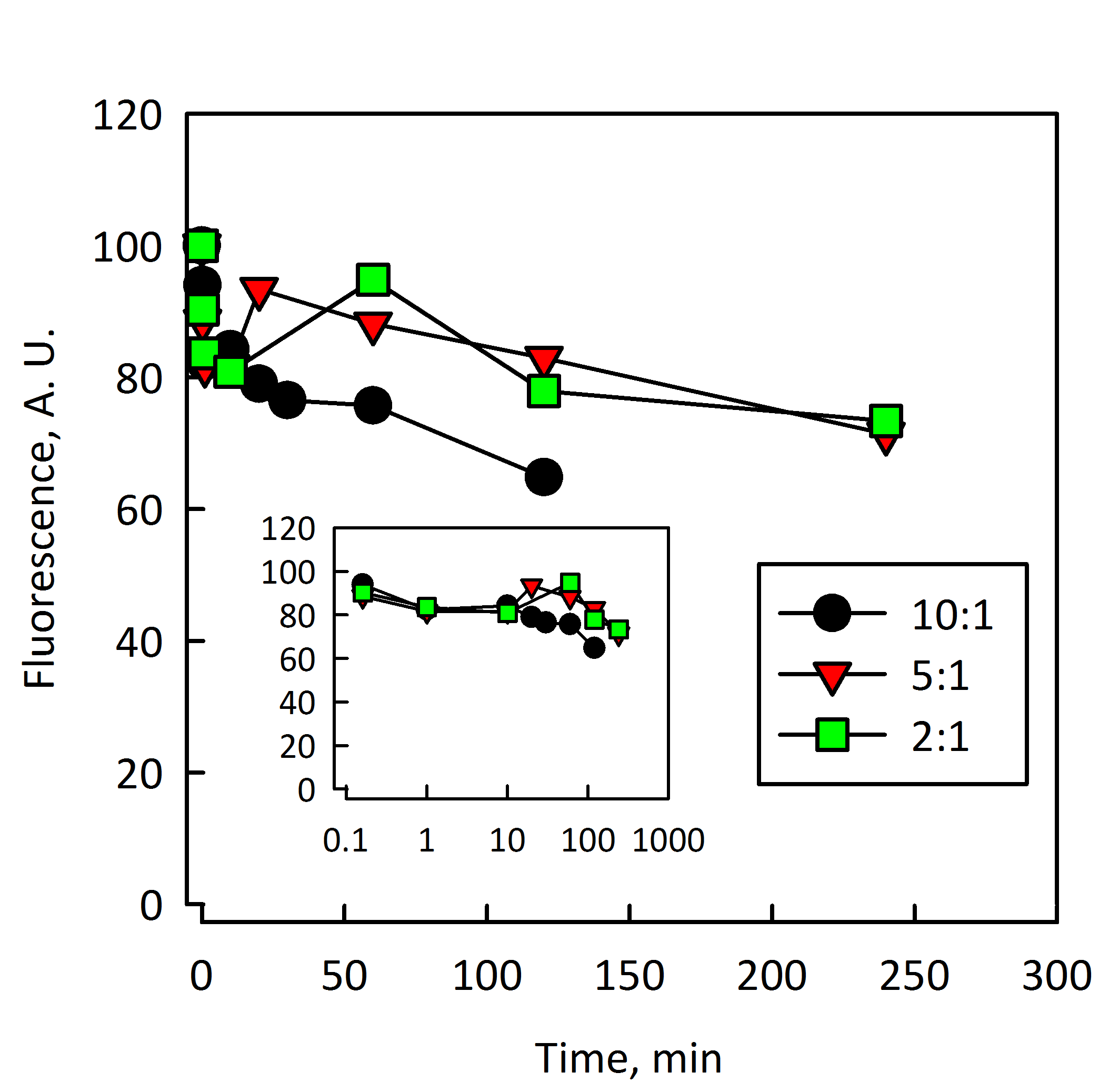

Supplement: S3 Fig — Human amylin (5 mg/mL) was incubated at 25°C in DMSO with varying amount of mPEGsc5k and the kinetics followed by quantifying the remaining primary amines with fluorescamine (10 μL reaction milieu + 200 μL fluorescamine 0.5 mg/mL in DMSO + 200 μL PBS, followed by immediate fluorescence reading). Inset: log scale. (TIF) [file pone.0138803.s003.tif]
