# Supplementary material for: Monoconjugation of Human Amylin with Methylpolyethyleneglycol
Source: PLoS One. 2015 Oct 8;10(10):e0138803. doi: 10.1371/journal.pone.0138803 (PMC4598023; doi:10.1371/journal.pone.0138803)
Supplement: S1 File — a) Human CTR1 Extracellular Topological Domain (ETD) -pET28b. b) Human RAMP3 Extracellular Topological Domain (ETD)—pET28b. (PDF) [file pone.0138803.s004.pdf]

S1 File. The aminoacid sequence of the constructs used in this work

a) Human CTR1 Extracellular Topological Domain (ETD) -pET28b
GSSHHHHHHSSGLVPRGSHMENLYFQGAFSNQTYPTIEPKPFLYVVGRKKMMDAQYKC
YDRMQQLPAYQGEGPYCNRTWDGWLCWDDTPAGVLSYQFCPDYFPDFDPSEKVTKYC
DEKGVWFKHPENNRTWSNYTMCNAFTPEKLKNAYVLYY

b) Human RAMP3 Extracellular Topological Domain (ETD) - pET28b
GSSHHHHHHSSGLVPRGSHMCNETGMLERLPLCGKAFADMMGKVDVWKWCNLSEFIV
YYESFTNCTEMEANVVGCYWPNPLAQGFITGIHRQFFSNCTVDRVHLEDPPDE
