# Supplementary material for: Monoconjugation of Human Amylin with Methylpolyethyleneglycol
Source: PLoS One. 2015 Oct 8;10(10):e0138803. doi: 10.1371/journal.pone.0138803 (PMC4598023; doi:10.1371/journal.pone.0138803)
Supplement: S1 Table — a) Two tailed p value. Insulin: hAmylin-PEG versus Insulin. b) Two tailed p value. Insulin:hAmylin-PEG versus Insulin:mAmylin. c) Two tailed p value. Insulin:hAmylin-PEG versus Insulin:hAmylin. (PDF) [file pone.0138803.s006.pdf]

S1 Table. Statistical Analysis of the Pharmacologic Evaluation – Glycemia.

a) Two tailed p value. Insulin: hAmylin-PEG *versus* Insulin

**Time**	**P value**	**Sign**
1h	P= 0.0144	*
2h	P= 0.437	-
4h	P= 0.00666	**
6h	P= 0.02	*
20h	P= 0.482	-

b) Two tailed p value. Insulin hAmylin-*PEG* *versus* Insulin:mAmylin

**Time**	**P value**	**Sign**
1h	P= 0.314	-
2h	P= 0.781	-
4h	P= 0.0174	*
6h	P= 0.0297	*
20h	P= 0.260	-

c) Two tailed p value. Insulin:hAmylin-PEG *versus Insulin*:hAmylin

**Time**	**P value**	**Sign**
1h	P= 0.125	-
2h	P= 0.2	-
4h	P= 0.0871	-
6h	P= 0.341	-
20h	P= 0.927	-
